# Quality assessment of adult intensive care services: application of a tool adjusted to the reality of low-income countries

**DOI:** 10.5935/0103-507X.20190031

**Published:** 2019

**Authors:** Alexandre Guilherme Ribeiro de Carvalho, Ana Paula Pierre de Moraes, Ana Cláudia Pinho de Carvalho, Antônio Augusto Moura da Silva

**Affiliations:** 1 Departamento de Saúde Pública, Universidade Federal do Maranhão - São Luís (MA), Brasil.; 2 Serviço de Terapia Intensiva, UDI Hospital - São Luís (MA), Brasil.; 3 Serviço de Terapia Intensiva, Hospital Tarquínio Lopes Filho - São Luís (MA), Brasil.

**Keywords:** Structure of services, Process assessment (health care), Outcomes, Intensive care units, Estrutura de serviços, Avaliação de processos (cuidados de saúde), Resultados, Unidades de terapia intensiva

## Abstract

**Objective:**

To assess the quality of adult intensive care units.

**Methods:**

This population-based, cross-sectional, observational, analytical study evaluated management type in Maranhão, Brazil. An assessment instrument was applied that assigned scores to each service (maximum 124 points). The units were categorized as insufficient (< 50% of the maximum score), typical (≥ 50% and <80% of the maximum score), or sufficient (≥ 80% of the maximum score).

**Results:**

Of the 26 intensive care units in Maranhão, 23 were evaluated; 15 (65.2%) were located in the state capital, and 14 (60.9%) were public. The mean final score was 67.2 (54.2% of the maximum). The worst performance was observed with regard to processes (50.9%) in the units located outside the capital (p = 0.037) and for hospitals with 68 beds or fewer (p = 0.027). The result of the assessment categorized services as a function of the overall total points earned. Specifically, 8 (34.8%) services were assessed as insufficient, 13 (56.5%) were assessed as typical, and 2 (8.7%) were assessed as sufficient.

**Conclusion:**

The majority of the intensive care units in this study were assessed as typical. These services must be better qualified. The priorities are the processes of the units located outside the capital and in small hospitals.

## Abstract (pt)

**Objetivo:**

Avaliar a qualidade de unidades de terapia intensiva adulto.

**Métodos:**

Estudo populacional, transversal, observacional, analítico, do tipo avaliação para gestão, no Estado do Maranhão. Um instrumento de avaliação foi aplicado, atribuindo pontuações para cada serviço (máximo 124). As unidades foram categorizadas como insuficientes (< 50% da pontuação máxima), regulares (≥ 50% e < 80% da pontuação máxima) ou suficientes (≥ 80% da pontuação máxima).

**Resultados:**

Das 26 unidades de terapia intensiva do Estado, 23 foram avaliadas; 15 (65,2%) estavam localizadas na capital, e 14 (60,9%) eram públicas. A pontuação final média foi de 67,2 (54,2% do máximo possível). O pior desempenho ocorreu nos processos (50,9%), nas unidades fora da capital (p = 0,037) e em hospitais com número de leitos ≤ 68 (p = 0,027). O resultado da avaliação consistiu na categorização dos serviços em função do total geral de pontos alcançados, a saber: 8 (34,8%) serviços receberam avaliação insuficiente, 13 (56,5%) regular e 2 (8,7%) suficiente.

**Conclusão:**

A maioria das unidades do estudo recebeu avaliação regular. Tais serviços necessitam ser melhor qualificados. As prioridades são os processos de unidades localizadas fora da capital e em hospitais de pequeno porte.

## INTRODUCTION

The expansion of intensive care services should be aligned with the quality of the services provided. Assessment studies might help to improve care and outcomes.^(1)^

Care quality assessment studies took many years to gain acceptance in Brazil; they began to gain popularity in the 1990s, but the number of studies increased only after 2004.^(2,3)^ The majority of studies focused on health programs and services in primary care, primarily in the public sector. Some programs and services, especially those in the hospital setting, were left out of the assessment process.^(4)^ Consequently, few national scientific quality assessments in the field of intensive medicine exist.^(2)^ This lack of intensive care quality assessments runs counter to the normative guidelines, the needs of the Unified Health System (Sistema Único de Saúde - SUS), and the importance of these services within the healthcare system, especially in light of the amount of funds allocated to intensive care services because they combine knowledge, technologies, and diagnostic and therapeutic methods across various healthcare fields.^(5,6)^

Despite the ongoing need to discuss topics on the current agenda such as safety, effectiveness, and costs/benefits, the currently available preclude the accurate determination of whether the quality of the adult intensive care units (ICUs) in Brazil is adequate. The present study hypothesizes that these units have shortcomings related to their structure, process systematization, and outcome achievement.

The current study evaluated adult ICUs with regard to their structure, processes, and outcomes.

## METHODS

A population-based, cross-sectional, observational, analytical study was conducted to evaluate management type.^(7)^

The study was conducted in the adult ICUs of Maranhão, Brazil. These ICUs were identified using the following secondary databases: the 2010 Census of the *Associação Brasileira de Medicina Intensiva Brasileira* (AMIB) and the 2014 *Cadastro Nacional de Estabelecimento de Saúde* (CNES). Population data from the *Institute Brasileiro de Geografia and Estatística* (IBGE) projected for 2014 were also used.^(8)^

Intensive care units intended for adults (i.e., ≥ 15 years old) were considered as eligible.^(9)^

Data were collected between January and March 2014. First, the coordinators of the services were contacted to schedule the data collection. During this contact, they were informed that on the day of the visit, the presence of the medical, nursing, and physical therapy coordinators would be necessary, as would access to the following data: the standardized mortality rate, the unplanned extubation rate, the mean length of ICU stay (in days), the ICU readmission rate, the mechanical ventilation-associated pneumonia rate, the density of catheter-related bloodstream infection, and the catheter-related urinary tract infection rate.

The data were collected by completing an assessment instrument (Appendix 1 - Supplementary material) specifically designed to address the conditions of Brazil and low-income countries.^(10)^ In this instrument, the standard considered for each investigated attribute was its presence at or above the minimum cutoff point. When an attribute was not measured or absent, it was classified as "out of standard". The assessment instrument also included a third criterion (below the standard) for attributes that were present or measured but scored below the minimum cutoff point established by the standard. The criteria were scored thusly: out of standard, 0; below standard, 1; standard, 2. At the end of the data collection period, the total score of each service was calculated, as was their performance with regard to the various dimensions investigated (Table 1). Regarding the outcome dimension, the available responses were coded as "9 - Not reported" for cases in which the indicator was measured and available for consultation but could not be provided to the interviewer due to institutional policy (e.g., confidential data). These cases were excluded from the overall score and did not part of the final sum of the service's score.

**Table 1:** Scores of the various dimensions, number of indicators, and maximum values on the quality assessment instrument

Dimension	Number of indicators	Maximum score
Structure	38	76
Physical structure	4	8
Human resources	14	28
Continuing education and training	2	4
Protocols and routines	12	24
Material resources	6	12
Process	17	34
Safety	7	14
Work	10	20
Outcomes	7	14
Final score	62	124

The evaluation tool was completed by the investigators during face-to-face interviews conducted at the service based on the answers obtained from the medical, nursing, and physical therapy coordinators of the participating ICUs.

The indicators collected included a description of the hospital and participating ICU (name, city, entity funding the hospital, main source of payment for ICU admissions, number of beds for general and ICU admissions, the existence of a professional training program, the nature of the services provided, and type of care), 38 structure indicators (four regarding physical structure, 14 regarding human resources, two regarding continuing education and training, 12 regarding protocols and routines, and six regarding material resources), 17 process indicators (seven regarding safety processes and 10 regarding work processes), and seven outcome indicators, totaling 62 indicators and 124 possible points.

The result of the ICU assessment was categorized as a function of the overall total score: up to 61 points (< 50% of the total possible points), insufficient assessment; 62 to 98 points, typical assessment; and ≥ 99 points (≥ 80% of the total possible points), sufficient assessment. This categorization was based on prior health service assessment studies.^(11,12)^

The indicators were also individually categorized to identify the topics with the greatest need for improvement. As such, the percentage of ICUs in compliance with the standard for each indicator was considered: if < 50% of the ICUs had reached the standard as specified (indicator within or above the minimum cutoff point), then the indicator was categorized as insufficient; 50% to 79% of the standard was classified as typical; and ≥ 80% of the standard was categorized as sufficient.

The mean overall and by section scores were compared according to the ICU location (capital or interior), the source of funding (public or private), the existence of a training program (yes or no), the ICU size (>10 or ≤ 10 beds) being the cutoff point based on the first quartile of the number of ICU beds per hospital, and the hospital number of beds (>68 or ≤ 68 beds) being the cutoff point established based on the first quartile of the total number of beds per hospital.

Dichotomous variables were expressed as frequencies and percentages. The Shapiro-Wilk test was used to examine the distribution of continuous variables. Continuous variables with normal distributions were expressed as means and standard deviations, and those without normal distributions were expressed as medians and interquartile ranges. The variances between groups of continuous variables was compared using the *F*-test. Student's *t-*test was used to compare the mean scores. Spearman's rank correlation coefficient was used to evaluate the correlation between the number of hospital beds and ICU beds as well as the score of the sections (structure, processes, and outcome) and the total score. For all analyses, 95% confidence intervals (95%CI) were calculated. A p-value ≤ 0.05 was considered as significant. STATA version 10.0 was used for all analyses.

The Research Ethics Committee of the University Hospital of the *Universidade Federal do Maranhão* approved the study under opinion number 289.199. The individuals responsible for the participating ICUs signed an authorization form for research participation. All participants signed an informed consent document.

## RESULTS

During the study period, Maranhão had 26 eligible adult ICUs, distributed among 22 hospitals in five municipalities: São Luís, Imperatriz, Caxias, Presidente Dutra, and Coroatá. Of these ICUs, three (11.5%; all from São Luís and privately funded) did not agree to participate in the study. The final sample included 23 ICUs: 15 (65.2%) were in the capital (10 public, four private, and one mixed) and eight (34.8%) were in rural cities (four private and one public in Imperatriz and one public each in Caxias, Presidente Dutra, and Coroatá).

The sample encompassed 333 beds allocated to adult ICUs (8.6% of the total of 3,881 hospital beds); however, only 320 were active (96.1% of the total). The ICU beds:inhabitants ratio in Maranhão was 1:20,573, considering only the ICUs that participated in this study. The median ICU beds per hospital was 12 (interquartile range, 25 - 75%: 10 to 20 beds), and the total number of hospital beds per hospital was 120 (25 - 75%: 68 to 195 beds).

Regarding hospital funding, only one (4.4%) had a mixed budget (public and private). The main sources of payment were the SUS for admission into 19 ICUs (82.6%) and private insurance for admission into three ICUs (13%).

The main reasons for admission were trauma in five ICUs (21.7%), surgery in three (13%), heart disease in two (8.8%) and cancer in one (4.4%). Twelve (52.2%) of the services were general units.

The maximum possible scores, both per section and the overall total, are presented in table 2. The mean total score was 67.2, which corresponds to 54.2% of the maximum points possible (124); the worst performance was found with regard to the process section (50.9% of the total possible score).

**Table 2:** Mean scores and percent of the maximum score, both total and per evaluated section, of the adult intensive care units in Maranhão

	Mean (SD)	Maximum score (%)
Structure	41.8 (12)	55.0
Processes	17.3 (7.4)	50.9
Outcomes	8.1 (3.5)	57.9
Overall Total	67.2 (21.5)	54.2

SD - standard deviation

Table 3 shows data relating to the ICU, the hospital, and its funding. Intensive care units located outside the capital and those in hospitals with fewer than 68 beds had significantly lower scores. Intensive care units in hospitals with vocational training programs showed high scores, but this difference was not significant.

**Table 3:** Overall total score and scores per section of the adult intensive care units in Maranhão

	n (%)	Overall score Mean ± SD (%)	p value	Structure score Mean ± SD (%)	p value	Process score Mean ± SD (%)	p value	Outcomes score Mean ± SD (%)	p value
Location									
Capital	15 (65.2)	73.9 ± 21.4 (59.6)	0.037	45.1 ± 12.1 (59.3)	0.067	19.5 ± 6.9 (57.4)	0.048	9.3 ± 3.7 (66.4)	0.013
Interior	8 (34.8)	54.6 ± 16.1 (44)		35.5 ± 9.7 (46.7)		13.1 ± 6.7 (38.5)		6 ± 2.1 (42.9)	
Hospital funding entity									
Private	8 (34.8)	67.6 ±30.8 (54.5)	0.95	41.2 ± 17.7 (54.2)	0.885	17.9 ± 9.6 (52.6)	0.759	8.4 ± 4.5 (60)	0.772
Public	14 (60.9)	66.9 ± 14.1 (54)		42.1 ± 7.2 (55.4)		16.9 ± 5.9 (49.7)		7.9 ± 2.9 (56.4)	
Existence of a vocational training program									
Yes	9 (39.1)	75.8 ± 22.6 (61.1)	0.142	46.2 ± 11.4 (60.8)	0.162	20.4 ± 8.8 (60)	0.099	9.1 ± 3.7 (65)	0.307
No	14 (60.9)	61.6 ± 19.6 (49.7)		38.9 ±- 12 (51.2)		15.2 ±5.7 (44.7)		7.5 ± 3.5 (53.6)	
Number of ICU beds*									
> 10	15 (65.2)	69.4 ± 25.1 (56)	0.509	42.8 ± 13.6 (56.3)	0.594	18.3 ± 8.2 (53.8)	0.321	8.3 ± 4.3 (59.3)	0.686
≤ 10	8 (34.8)	63 ± 12.7 (50.8)		39.9 ± 8.9 (52.5)		15.4 ± 5.4 (45.3)		7.8 ± 1.4 (55.7)	
Number of hospital beds†									
> 68	17 (73.9)	72.9 ± 19.7 (58.8)	0.027	45.1 ± 10.4 (59.4)	0.058	18.8 ± 7.6 (55.3)	0.335	9.1 ± 3.3 (65)	0.039
≤ 68	6 (26.1)	50.8 ± 18.9 (41)		32.5 ± 12.4 (42.8)		12.8 ± 4.4 (37.6)		5.5 ± 3.1 (39.3)	

SD - standard deviation; ICU - intensive care unit;

* cutoff established based on the first quartile of the number of intensive care unit beds per hospital;

† cutoff point established based on the first quartile of the total number of beds per hospital.

According to the criterion adopted by this study, eight (34.8%) ICUs were assessed as insufficient, 13 (56.5%) were assessed as typical, and two (8.7%) were assessed as sufficient. Spearman's rank correlation coefficient showed significant correlations between the total scores earned in the structure and processes sections (*r =* 0.79; p < 0.001), the structure and outcomes sections (*r =* 0.68; p < 0.001), and the processes and outcomes sections (*r =* 0.69; p < 0.001).

Table 4 shows the classification of the indicators according to the percentage of ICUs that complied with the standard criteria. Three (4.8%) indicators were identified as sufficient (one for structure and two for processes), 21 (33.9%) were identified as typical (15 for structure, three for processes, and three for outcomes), and 38 (61.3%) were identified as insufficient (22 for structure, 12 for processes, and four for outcomes).

**Table 4:** Classification of indicators according to the standard criteria of the adult intensive care units in Maranhão

Indicator	%
**Sufficient**	
Structure	
Ratio of nursing technicians per bed per shift (relative to the shift with the poorest ratio)	87.0
Processes	
Multi-professional notes of the care provided in the ICU are recorded in the patients’ clinical records.	100.0
Multidisciplinary (bedside or rounds-style) discussions of current cases are performed in the ICU.	87.0
**Typical**	
Structure	
Medical technical manager is an accredited specialist in adult intensive care medicine	78.3
Ratio of nurses on duty per bed per shift (relative to the shift with the poorest ratio)	78.3
Availability of a medical technical manager in the ICU	73.9
Availability of a nursing coordinator in the ICU	73.9
Availability of a written protocol or routine for glycemic control	69.6
Availability of a written protocol or routine for standard preventive and transmission-based (contact, droplets, and aerosols) preventive measures	65.2
Availability of a written protocol or routine to prevent ventilator-associated pneumonia (mark "yes" when unit uses "bundles")	60.9
Availability of a written protocol or routine for the use of antibiotics	60.9
Physical therapy coordinator participated in a specialization course or is accredited in intensive physical therapy	56.5
Availability of a written protocol or routine with the criteria for admission to and discharge from the unit	56.5
Availability of a written protocol or routine for sedation	56.5
Availability of a written protocol or routine to prevent venous thromboembolism	56.5
Availability of a written protocol or routine for pain management	52.2
Availability of a written protocol or routine to prevent catheter-related bloodstream infection (mark "yes" when the unit uses "bundles")	52.2
Processes	
ICU requires a signature on an informed consent form for the procedures most frequently performed in the ICU	65.2
ICU monitors adverse and sentinel events	52.2
Periodicity of revisions made to protocols and routines	52.2
Outcomes	
ICU readmission rate over the past 12 months (or other available period of time)	69.6
Rate of catheter-related bloodstream infection (CRBI) over the past 12 months (or other available period of time)	69.6
Rate of ventilator-associated pneumonia (VAP) over the past 12 months (or other available period of time)	60.9
**Insufficient**	
Structure	
Availability of the waiting room for attendants and visitors	47.8
Ratio of physicians on duty per bed per shift (relative to the shift with the poorest ratio)	47.8
Availability of and regular participation in a continued education program for the multi-professional staff (doctors, nurses, and physical therapists) after assignment to the unit	47.8
Availability of a written protocol or routine for the use of blood components	47.8
Availability of a written protocol or routine for a lung-protective ventilatory strategy	47.8
Availability of written protocol or routine for gastrointestinal bleeding caused by stress	47.8
Availability of an electrocardiography device	43.5
Availability of isolation beds	39.1
Nursing coordinator participated in a specialization course or is accredited in intensive care nursing	39.1
Availability of a physical therapy coordinator in the ICU	30.4
Ratio of regular attending physicians per bed per shift (relative to the shift with the poorest ratio)	30.4
Ratio of physical therapists per bed per shift (relative to the shift with the poorest ratio):	26.1
Availability of a crash cart	26.1
Availability of a defibrillator/cardioverter	26.1
Availability of a transport ventilator	26.1
Daily availability of regular attending physicians in the ICU	21.7
Regular attending physicians are accredited specialists in intensive care medicine	17.4
Availability of a temporary transvenous cardiac pacing generator	17.4
Availability of a room for interviews with relatives or other attendants	13.0
Availability of a clinical engineering service at the hospital	8.7
Availability of clocks and calendars visible from all of the beds	8.7
Availability of a systematized and regular ICU-centered training program for professionals at the institution before assignment to the unit (e.g., integration programs)	4.4
Processes	
HICC provides the ICU multi-professional staff reports on the consolidated results of infection surveillance and the sensitivity profile of microorganisms	43.5
Periodicity of multidisciplinary (bedside or rounds-style) discussions of current cases	43.5
HICC participates in (bedside or rounds-style) multidisciplinary discussions of current cases at ICU	43.5
Visitors and attendants are given orientation to actions that will facilitate the prevention and control of infections based on the Hospital Infection Control Committee’s (HICC’s) recommendations	39.1
ICU performs a systematized analysis of adverse and sentinel events using standardized tools aimed at the identification of their causes and the elaboration of preventive strategies	30.4
ICU performs evaluations using a system of classification of nursing care needs (e.g., TISS, NAS, and Fugulin)	30.4
ICU monitors and evaluates its technical-operational performance	26.1
ICU and HICC provide joint training to improve the adherence of the multi-professional staff to routine hand washing	17.4
ICU communicates to the multi-professional staff the results of the monitoring and evaluation of its technical-operational performance	17.4
ICU assesses the satisfaction of patients and relatives	17.4
ICU conducts prescheduled meetings with relatives or attendants of patients to provide information on their state of health and the care they need (do not consider information provided during regular visiting times)	4.4
Relatives or attendants of patients can stay in the ICU	4.4
Outcomes	
Rate of catheter-associated urinary tract infections (CA-UTI) over the past 12 months (or other available period of time)	39.1
Unplanned extubation rate over the past 12 months (or other available period of time)	34.8
Standardized mortality rate over the past 12 months (or other available period of time)	17.4
Average length of stay in the ICU, in days, over the past 12 months (or other available period of time):	17.4

ICU - intensive care unit; HICC - hospital infection control committee; TISS - Therapeutic Intervention Score System; NAS - Nursing Activity Score.

## DISCUSSION

The results of the present study indicate a performance considered as typical for adult ICUs in Maranhão. Consequently, priorities and public policies to modify the current reality should be developed with greater focus and foundation. Importantly, despite the major regulatory advance established by the Board of Directors Resolution number 7 of 2010,^(9)^ a long road must be traveled before it is fully implemented throughout Brazil.

The systematic data collection regarding the quality of healthcare services, the evolutionary comparison of the information produced, and the subsequent application of new assessments are the pillars for improvements in healthcare provision.^(13)^ As Brook et al. highlighted, significant improvements in population health might be obtained by using the best currently available knowledge rather than new evidence or technologies yet to be discovered.^(14)^

This study is the first systematic approach that sought to objectively assess the ICUs of Maranhão. According to Machado et al., only two assessments of ICU quality had been published until 2011.^(2)^ Both studies only investigated outcome indicators (i.e., length of stay in the unit, length of hospital stay, and hospital mortality).^(15,16)^

This study has specific limitations such as its small sample size, which decreases the power for finding significant differences. However, the study employed a population-based sample because it examined ICUs in Maranhão. Three of the 26 adult ICUs did not agree to participate in the study, which is another limitation. The occurrence of selection bias is unlikely because the percentage of losses was low. One possible source of information bias is that a structured form was applied during an interview to collect data. To reduce this bias, the assessment instrument prioritized objectives that are easy to collect. Another limitation was the single focus evaluation technique (i.e., the application of a structured form), and other data collection techniques were not used (e.g., field observations, review of medical records, interviews with system users, and so on). Even with a single approach, however, valid and reliable information can be produced with the added advantage of a lower cost.^(14)^ In addition, the assessment tool used was not considered as the gold standard.

However, the study was able to collect relevant information aligned with the reality of healthcare practices because it used an evaluation instrument appropriate to the peculiarities of Brazil; employed structure and process indicators that are correlated with results; and identified opportunities for improvement and priorities for action, enabling self-assessments of the service over time and policies based on the reality of healthcare.

A 2007 study of 40 French ICUs also used scores to assess the quality of services. The median total score corresponded to 55% of the maximum possible score, a value similar to that of the present study (54.2%).^(6)^ However, a comparison of service assessments that used different data collection instruments might be inconclusive. The theoretical and methodological bases of the evaluations assume that the construct "quality" is defined *a priori* and focuses on the interest of the authors.^(17)^

The results of this study are of great interest to the discussions concerning improvements to intensive care services. The first is that quality did not significantly differ based on the criteria defined herein between the public and private sectors with regard to all dimensions evaluated, including in relation to structural aspects. The second is that the ICUs located outside the capital and allocated to hospitals with fewer than 68 beds performed worse on the processes and outcomes sections. These data suggest a worrying scenario. The majority of the state's population is concentrated in the interior regions of Maranhão, which have the worst Human Development Indices and unfavorable health indicators.^(8,18)^ These locations have fewer public hospitals, available ICU beds, and qualified services. American hospitals with better technical performance are those of medium/large size located in urban areas, corroborating the results of our study.^(18)^

Given the ICUs that participated in this study, the ratio of ICU beds per inhabitants in Maranhão was 0.5 per 10,000 inhabitants, a number below other regions of the country.^(19)^ These data reinforce the need for governmental efforts, both regarding the qualification and the expansion of ICUs in Maranhão.

The data regarding inadequate medical team size deserve attention. Studies have shown that the 24-hour presence of a medical specialist in the ICU is associated with lower mortality rates and lengths of stay.^(20,21)^ In the present study, one third of the ICUs did not have attending physicians. In two-thirds of the ICUs that did have such a professional, this individual was not trained in intensive medicine. The ratio of physicians on duty per bed was inadequate in more than half of the ICUs visited. Programs for professional training in ICU work were not available in almost all of the units prior to admission.

Almost half of the ICU did not monitor adverse events. This finding stems from the lack of culture and instruments used for the systematic performance of this process. However, evidence has shown that this approach is useful for assessing quality of care as well as improving the safety of care and communication among the multidisciplinary team.^(22)^

The systematic evaluation of customer satisfaction as well as the greater presence of family members positively affect the quality of care provided. These interventions are simple and inexpensive, whose implementation should be considered for ICUs. The perspective of healthcare system users has been cited as an important indicator in the planning of improvement actions.^(17,23)^ In Maranhão, only one quarter of ICUs have conducted user satisfaction surveys.

One positive finding of this study was the high percentage of ICUs that conducted multidisciplinary discussions because these conversations are associated with reduced mortality rates.^(21,24,25)^ The implementation of this process represents little or no additional cost for the institution and is a high-impact measure that can be adopted by intensive care services.

The routine measurement of unit outcomes, in addition to being established in the regulations for the sector,^(26)^ enables the construction of time series and the monitoring of the effect caused by the interventions and improvements made.^(14)^ Ultimately, the monitoring actions themselves improve the service's performance, a phenomenon known as the Hawthorne effect.^(27)^ In this study, almost half of the services visited did not record basic data (e.g., mortality adjusted for patient severity). We emphasize that the clinical characteristics (case mix) of the hospitalized patients also strongly influence the outcomes achieved.

We emphasize that the results were typical regarding the indicators related to the surveillance of healthcare-associated infections (HAIs). However, this finding might have been caused by the inclusion of excessively sensitive cutoff points because no large-scale studies of national ICUs have measured the prevalence of HAIs.

The correlation between the score of the structure and processes sections and the score of the outcomes section confirms the findings of other studies^(21,25,28-31)^ and suggests that the modifications and improvements to be implemented in the first two sections have the potential to improve the outcomes of the services that participated in the study.

Based on the data generated and the weaknesses identified, improvements related to the indicators with poorer performance should be discussed and implemented. The processes of the ICUs allocated to smaller hospitals in the interior of Maranhão are priorities for intervention. The main actions of the discussion agenda shown by the current study are structural projects that prioritize the comfort and humanization of units in parallel with the development of patient/user-centered care processes; adjustments to the number and training of human resources; stimulus for the elaboration, dissemination, training, implementation, and measurement of the results of national protocols for interventions of greater risk or benefit based on available evidence; the development of checklist-type tools for the main processes performed in intensive care services to increase adherence;^(21,32)^ funding and tax incentives for equipment acquisition; the development of tools with user-friendly interfaces to analyze events and user satisfaction; incentives for the development of intensive care medicine to elaborate career plans and specific job openings for these professionals; differentiated resource transfer policies that consider the results of the quality assessment of services, not only their operational results;^(33)^ increased interactions among the services of large centers and those of smaller locations, both in person and through available technology (e.g., videoconferencing);^(21)^ the production and use of assessment tools and studies; and manager training.

Ultimately, investments in assessment and the qualification of healthcare services are fully justifiable. In addition to their transforming actions with consequent increases in efficiency as well as the social and constitutional functions of promoting and recovering health, better qualified services are less expensive.^(34)^

## CONCLUSION

Most intensive care services in Maranhão were assessed as typical. These services must be better qualified and expanded. In particular, priority should be given to the processes used in intensive care units at smaller hospitals located outside the state capital.
