# Supplementary material for: Quality assessment of adult intensive care services: application of a tool adjusted to the reality of low-income countries
Source: Rev Bras Ter Intensiva. 2019 Apr-Jun;31(2):138–46. doi: 10.5935/0103-507X.20190031 (PMC6649218; doi:10.5935/0103-507X.20190031)
Supplement: Supplementary file 1 [file rbti-31-02-0138-suppl1.pdf]

Quality assessment of adult intensive care services: application of a tool adjusted to the reality of low-income countries

Avaliação da qualidade de serviços de medicina intensiva adulto: aplicação de ferramenta ajustada à realidade de países de baixa renda

Alexandre Guilherme Ribeiro de Carvalho[1,2], Ana Paula Pierre de Moraes[3], Ana Cláudia Pinho de Carvalho[2], Antônio Augusto Moura da Silva[1]

Appendix 1 - Assessment instrument

SECTION A: HOSPITAL AND ICU DATA	
1 - Hospital:	
2 - Town/State:	
3 - Hospital funding mechanism:	( ) Public ( ) Private ( ) Mixed ( ) Philanthropic ( ) Military
4 - Number of inpatient beds (exclude beds for observation and "day-hospital"):	
5 - Availability of a training program at the ICU or hospital (e.g., graduate or residency program):	( ) 0 No ( ) 1 Yes
6 - University hospital:	( ) 0 No ( ) 1 Yes
7 - Number of ICUs in the hospital:	
8 - Identity of the participating ICU:	
9 - Main source of payment for ICU admissions (mark the option that represents the source accounting for most payments for ICU admissions):	( ) 1 Public health system ( ) 2 Health insurance companies<br>( ) 3 Private ( ) 4 Other
10 - Type of ICU (mark the option that represents the ICU's main focus or the most prevalent type of patients):	( ) 1 General ( ) 2 Cardiological ( ) 3 Surgical ( ) 4 Neurological<br>( ) 5 Trauma ( ) 6 Oncological ( ) 7 Other
11 - Total number of beds in the ICU:	
12 - Total number of beds currently functioning at the ICU:	
SECTION B: STRUCTURE INDICATORS	
**B.1 - Physical structure**	
13 - Availability of isolation beds:	( ) 0 No<br>( ) 1 Yes, ratio < 1:10 beds<br>( ) 2 Yes, ratio ≥ 1:10 beds
14 - Availability of a room for interviews with relatives or other attendants:	( ) 0 No<br>( ) 1 Yes, multifunctional, not exclusively for this purpose<br>( ) 2 Yes, exclusively for this purpose
15 - Availability of the waiting room for attendants and visitors:	( ) 0 No<br>( ) 1 Yes, shared with other services<br>( ) 2 Yes, exclusively for the ICU
16 - Beds allow for patient privacy when needed (e.g., during baths); obstetrics: exclude screens:	( ) 0 No<br>( ) 1 Yes, in some beds<br>( ) 2 Yes, in all beds
**TOTAL SCORE FOR B.1 (MAXIMUM: 8):**	
**B.2 - Human resources**	
17 - Availability of a clinical engineering service at the hospital:	( ) 0 No ( ) 1 Yes, outsourced ( ) 2 Yes, in-house

continue...

... continuation

18 - Availability of a medical technical manager in the ICU:	( ) 0 No<br>( ) 1 Yes, shared with other hospital units<br>( ) 2 Yes, exclusive to the ICU
19 - Medical technical manager is an accredited specialist in adult intensive care medicine:	( ) 0 No ( ) 2 Yes
20 - Availability of a nursing coordinator in the ICU:	( ) 0 No<br>( ) 1 Yes, shared with other hospital units<br>( ) 2 Yes, exclusive to the ICU
21 - Nursing coordinator participated in a specialization course or is accredited in intensive care nursing:	( ) 0 No<br>( ) 1 No, but currently attending<br>( ) 2 Yes, already completed
22 - Availability of a physical therapy coordinator in the ICU:	( ) 0 No<br>( ) 1 Yes, shared with other hospital units<br>( ) 2 Yes, exclusive to the ICU
23 - Physical therapy coordinator participated in a specialization course or is accredited in intensive physical therapy:	( ) 0 No<br>( ) 1 No, but currently attending<br>( ) 2 Yes, already completed
24 - Daily availability of regular attending physicians in the ICU:	( ) 0 No<br>( ) 1 Yes, in at least one shift (day or night)<br>( ) 2 Yes, in the day and night shifts
25 - Regular attending physicians are accredited specialists in intensive care medicine:	( ) 0 No<br>( ) 1 Yes, some<br>( ) 2 Yes, all of them
26 - Ratio of regular attending physicians per bed per shift (relative to the shift with the poorest ratio):	( ) 0 Unavailable<br>( ) 1 < 1:10 beds<br>( ) 2 ≥ 1:10 beds
27 - Ratio of physicians on duty per bed per shift (relative to the shift with the poorest ratio):	( ) 0 < 1:10 beds<br>( ) 2 ≥ 1:10 beds
28 - Ratio of nurses on duty per bed per shift (relative to the shift with the poorest ratio):	( ) 0 Unavailable<br>( ) 1 < 1:10 beds<br>( ) 2 ≥ 1:10 beds
29 - Ratio of physical therapists per bed per shift (relative to the shift with the poorest ratio):	( ) 0 Unavailable<br>( ) 1 < 1:10 beds<br>( ) 2 ≥ 1:10 beds
30 - Ratio of nursing technicians per bed per shift (relative to the shift with the poorest ratio):	( ) 0 < 1:2 beds<br>( ) 2 ≥ 1:2 beds
**TOTAL SCORE FOR B.2 (MAXIMUM: 28):**	
**B.3 - Continued education and training**	
31 - Availability of a systematized and regular ICU-centered training program for professionals at the institution before assignment to the unit (e.g., integration programs):	( ) 0 No<br>( ) 1 Yes, but restricted to some professional categories<br>( ) 2 Yes, for doctors, nurses and physical therapists
32 - Availability of and regular participation in a continued education program for the multi-professional staff (doctors, nurses, and physical therapists) after assignment to the unit:	( ) 0 No or < 4 times per year<br>( ) 1 Yes, at least 4 times per year<br>( ) 2 Yes, every month

continue...

... continuation

**TOTAL SCORE FOR B.3 (MAXIMUM: 4):**	
**B.4 - Protocols and routines**	
33 - Availability of a written protocol or routine with the criteria for admission to and discharge from the unit:	( ) 0 No<br>( ) 1 Yes, but common to all the hospital ICUs or the hospital as a whole<br>( ) 2 Yes, specific to the unit
34 - Availability of a written protocol or routine for glycemic control:	( ) 0 No ( ) 2 Yes
35 - Availability of a written protocol or routine for pain management:	( ) 0 No ( ) 2 Yes
36 - Availability of a written protocol or routine for sedation:	( ) 0 No ( ) 2 Yes
37 - Availability of a written protocol or routine for the use of blood components:	( ) 0 No ( ) 2 Yes
38 - Availability of a written protocol or routine for a lung-protective ventilatory strategy:	( ) 0 No ( ) 2 Yes
39- Availability of a written protocol or routine for the prevention of ventilator-associated pneumonia (mark "yes" when unit uses "bundles"):	( ) 0 No ( ) 2 Yes
40 - Availability of a written protocol or routine for the prevention of catheter-related bloodstream infection (mark "yes" when the unit uses "bundles"):	( ) 0 No ( ) 2 Yes
41 - Availability of a written protocol or routine for the use of antibiotics:	( ) 0 No ( ) 2 Yes
42 - Availability of written protocol or routine for gastrointestinal bleeding caused by stress:	( ) 0 No ( ) 2 Yes
43 - Availability of a written protocol or routine for the prevention of venous thromboembolism:	( ) 0 No ( ) 2 Yes
44 - Availability of a written protocol or routine for standard preventive and transmission-based (contact, droplets, aerosols) preventive measures:	( ) 0 No<br>( ) 1 Yes, but formulated by the ICU or the HICC alone<br>( ) 2 Yes, formulated jointly by the ICU and the HICC
**TOTAL SCORE FOR B.4 (MAXIMUM: 24):**	
**B.5 - Material resources**	
45 - Availability of a electrocardiography device:	( ) 0 No or shared with other units<br>( ) 1 Yes, $<$ 1 per 10 beds<br>( ) 2 Yes, $\geq$ 1 per 10 beds
46 - Availability of a crash cart:	( ) 0 No or shared with other units<br>( ) 1 Yes, $<$ 1 per 5 beds<br>( ) 2 Yes, $\geq$ 1 per 5 beds
47 - Availability of a defibrillator/cardioverter:	( ) 0 No or shared with other units<br>( ) 1 Yes, $<$ 1 per 5 beds<br>( ) 2 Yes, $\geq$ 1 per 5 beds
48 - Availability of a temporary transvenous cardiac pacing generator:	( ) 0 No or shared with other units<br>( ) 1 Yes, $<$ 1 per 10 beds<br>( ) 2 Yes, $\geq$ 1 per 10 beds
49 - Availability of a transport ventilator:	( ) 0 No or shared with other units<br>( ) 1 Yes, $<$ 1 per 10 beds<br>( ) 2 Yes, $\geq$ 1 per 10 beds
50 - Availability of clocks and calendars visible from all of the beds:	( ) 0 No<br>( ) 1 Yes, from some beds<br>( ) 2 Yes, from all beds

continue...

... continuation

**TOTAL SCORE FOR B.5 (MAXIMUM: 12):**	
**TOTAL SCORE FOR SECTION B (B.1 + B.2 + B.3 + B.4 + B.5):**	
**MAXIMUM: 76**	
SECTION C: PROCESS INDICATORS	
**C.1 - Safety processes**	
51 - Visitors and attendants are given orientation to actions that will facilitate the prevention and control of infections based on the Hospital Infection Control Committee's (HICC's) recommendations:	( ) 0 No<br>( ) 1 Yes, at least verbally<br>( ) 2 Yes, verbally and in printed format
52 - ICU and HICC provide joint training to improve the adherence of the multi-professional staff to routine hand washing:	( ) 0 No<br>( ) 1 Yes, but not at established intervals<br>( ) 2 Yes, at established intervals
53 - HICC provides the ICU multi-professional staff reports on the consolidated results of infection surveillance and the sensitivity profile of microorganisms:	( ) 0 No or at intervals longer than three months<br>( ) 1 Yes, at least four times per year<br>( ) 2 Yes, every month
54 - ICU monitors adverse and sentinel events:	( ) 0 No ( ) 2 Yes
55 - ICU performs a systematized analysis of adverse and sentinel events using standardized tools aimed at the identification of their causes and the elaboration of preventive strategies:	( ) 0 No<br>( ) 1 Yes, but at intervals > 1 month<br>( ) 2 Yes, every month
56 - ICU monitors and evaluates its technical-operational performance:	( ) 0 No<br>( ) 1 Yes, but at intervals > 1 month<br>( ) 2 Yes, every month
57 - ICU communicates to the multi-professional staff the results of the monitoring and evaluation of its technical-operational performance:	( ) 0 No<br>( ) 1 Yes, but at intervals > 1 month<br>( ) 2 Yes, every month
**TOTAL SCORE FOR C.1 (MAXIMUM: 14):**	
**C.2 - Work processes**	
58 - Periodicity of revisions made to protocols and routines:	( ) 0 Are not revised or are unavailable<br>( ) 1 > 12 months<br>( ) 2 Once per year
59 - Multidisciplinary (bedside or rounds-style) discussions of current cases are performed in the ICU:	( ) 0 No<br>( ) 1 Yes, including doctors and nurses<br>( ) 2 Yes, including doctors, nurses and physical therapists at least
60 - Periodicity of multidisciplinary (bedside or rounds-style) discussions of current cases:	( ) 0 < 3 times per week<br>( ) 1 ≥ 3 and ≤ 6 times per week<br>( ) 2 Every day
61 - HICC participates in (bedside or rounds-style) multidisciplinary discussions of current cases at ICU:	( ) 0 No<br>( ) 1 Yes, but only upon request<br>( ) 2 Yes, at least once per week
62 - ICU conducts prescheduled meetings with relatives or attendants of patients to provide information on their state of health and the care they need (do not consider information provided during regular visiting times):	( ) 0 No<br>( ) 1 Yes, at least once during stay<br>( ) 2 Yes, more than once during stay
63 - ICU performs evaluations using a system of classification of nursing care needs (e.g., TISS, NAS, Fugulin):	( ) 0 No ( ) 2 Yes

continue...

... continuation

64 - Multi-professional notes of the care provided in the ICU are made in the patients' clinical records:	( ) 0 No
	( ) 1 Yes, but only doctors and nurses are allowed
	( ) 2 Yes, by doctors, nurses and physical therapists at minimum
65 - Relatives or attendants of patients can stay in the ICU:	( ) 0 No or only at visiting times
	( ) 1 Only at visiting times, which might be modified on an individual basis
	( ) 2 Free access 24/7
66 - ICU requires a signature on an informed consent form for the procedures most frequently performed in the ICU:	( ) 0 No ( ) 2 Yes
67 - ICU assesses the satisfaction of patients and relatives:	( ) 0 No
	( ) 1 Yes, but not routinely or in a systematized manner
	( ) 2 Yes, routinely and in a systematized manner
**TOTAL SCORE FOR C.2 (MAXIMUM: 20):**	
**TOTAL SCORE FOR SECTION C (C.1 + C.2):**	
**MAXIMUM: 34**	
SECTION D: OUTCOME INDICATORS	
68 - Standardized mortality rate in the past 12 months (or other available period of time):	( ) 0 Not measured
	( ) 1 > 1.0
	( ) 2 ≤ 1.0
	( ) 9 Not reported
69 - Unplanned extubation rate in the past 12 months (or other available period of time):	( ) 0 Not measured
	( ) 1 > 15%
	( ) 2 ≤ 15%
	( ) 9 Not reported
70 - Average length of stay in the ICU, in days, in the past 12 months (or other available period of time):	( ) 0 Not measured
	( ) 1 > 7 days
	( ) 2 ≤ 7 days
	( ) 9 Not reported
71 - ICU readmission rate in the past 12 months (or other available period of time):	( ) 0 Not measured
	( ) 1 > 10%
	( ) 2 ≤ 10%
	( ) 9 Not reported
72 - Rate of ventilator-associated pneumonia (VAP) in the past 12 months (or other available period of time):	( ) 0 Not measured
	( ) 1 > 16 cases per 1,000 days of mechanical ventilation
	( ) 2 ≤ 16 cases per 1,000 days of mechanical ventilation
	( ) 9 Not reported
73 - Rate of catheter-related bloodstream infection (CRBI) in the past 12 months (or other available period of time):	( ) 0 Not measured
	( ) 1 > 12 per 1,000 central line-days
	( ) 2 ≤ 12 per 1,000 central line-days
	( ) 9 Not reported
74 - Rate of catheter-associated urinary tract infections (CA-UTI) in the past 12 months (or other available period of time):	( ) 0 Not measured
	( ) 1 > 6 per 1,000 catheter-days
	( ) 2 ≤ 6 per 1.000 catheter-days
	( ) 9 Not reported
**TOTAL SCORE FOR SECTION D (MAXIMUM: 14):**	
**OVERALL TOTAL SCORE FOR SECTIONS B + C + D (MAXIMUM: 124):**	
